# Continuous adductor canal block versus continuous femoral nerve block for postoperative pain in patients undergoing knee arthroplasty: An updated meta-analysis of randomized controlled trials

**DOI:** 10.1371/journal.pone.0306249

**Published:** 2024-08-01

**Authors:** Jinyan Gong, Lu Tang, Yuyu Han, Pengcheng Liu, Xue Yu, Fei Wang

**Affiliations:** 1 Shandong University of Traditional Chinese Medicine, Jinan, China; 2 Department of Anesthesiology, the 960^th^ hospital of People’s Liberation Army of China (PLA), Jinan, China; 3 Department of Stomatology, the 960^th^ hospital of PLA, Jinan, China; 4 School of Anesthesiology, Weifang Medical University, Weifang, China; 5 Jinzhou Medical University, Jinzhou, China; Maulana Azad Medical College, INDIA

## Abstract

Continuous adductor canal block (CACB) is almost a pure sensory nerve block and can provide effective analgesia without blocking the motor branch of the femoral nerve. Thus, the objective of this study was to systematically evaluate the efficacy of CACB versus continuous femoral nerve block (CFNB) on analgesia and functional activities in patients undergoing knee arthroplasty. PubMed, Embase and the Cochrane Central Register of Controlled Trials (from inception to 3 October 2023) were searched for randomized controlled trials (RCTs) that compared CACB with CFNB in patients undergoing knee arthroplasty. Registration in the PROSPERO International prospective register of the meta-analysis was completed, prior to initiation of the study (registration number: CRD42022363756). Two independent reviewers selected the studies, extracted data and evaluated risk of bias by quality assessment. Revman 5.4 software was used for meta-analysis and the summary effect measure were calculated by mean differences and 95% confidence intervals. Eleven studies with a total of 748 patients were finally included. Pooled analysis suggested that both CACB and CFNB showed the same degree of pain relief at rest and at motion at 12 h, 24 h and 48 h in patients undergoing knee arthroplasty. Compared with CFNB, CACB preserved the quadriceps muscle strength better (P<0.05) and significantly shortened the discharge readiness time (P<0.05). In addition, there was no significant difference in opioid consumption, knee extension and flexion, timed up and go (TUG) test, or risk of falls between the two groups. Thus, Compared with CFNB, CACB has similar effects on pain relief both at rest and at motion and opioid consumption for patients undergoing knee arthroplasty, while CACB is better than CFNB in preserving quadriceps muscle strength and shortening the discharge readiness time.

## Introduction

Knee arthroplasty is a major orthopedic operation and has become a reliable means to treat serious knee diseases, relieve knee pain and rebuild knee function. However, it often leads to severe postoperative pain that may impair the rapid recovery of patients [1]. Inadequate pain relief is associated with peri-operative complications, difficult rehabilitation and prolonged recovery [2]. Therefore, effective analgesia is particularly important for patients undergoing knee arthroplasty.

Currently, multimodal analgesia is an ideal way to relieve pain for patients undergoing knee arthroplasty, such as epidural analgesia, patient-controlled opioid analgesia, oral analgesics and peripheral nerve block analgesia. These methods are often accompanied by a high incidence of adverse reactions, such as epidural hematoma caused by epidural analgesia, postoperative nausea and vomiting caused by opioids, and insufficient analgesic effect due to the first-pass elimination of oral analgesics. Peripheral nerve block has the advantages of exact analgesic effect, little interference to the internal environment, good tolerance of patients, and can reduce the dosage of analgesic drugs, so it has been widely used in postoperative analgesia. Femoral nerve block (FNB) is a commonly used modality for postoperative analgesia in patients undergoing knee arthroplasty [3, 4]. However, the disadvantage for FNB is that the quadriceps muscle strength essential for postoperative patient mobilization and early rehabilitation exercises is weakened, which may delay discharge from hospital [5–7]. In recent years, adductor canal block (ACB) as a new nerve block technique has been widely used in patients undergoing knee arthroplasty. The adductor canal lies medial to the front of the middle third of the femoris, deep to the sartorius and between the adductor magnus and the vastus medialis. The nerve structure in the adductor canal mainly includes the saphenous nerve and the medial branch of the femoral nerve, which are the main targets of ACB. Therefore, ACB is almost a pure sensory nerve block and can provide effective analgesia without blocking the motor branch of the femoral nerve. This retention of quadriceps strength without compromising the analgesic effect provided by ACB is supported by a number of studies [8–10]. Thus, ACB has been gaining favor as the preferred method of pain control for patients undergoing knee arthroplasty [11].

Because pain after knee arthroplasty usually outlasts the duration of a single-injection nerve block, a percutaneous perineural catheter is frequently inserted in order to allow prolonged local anesthetic administration to extend postoperative analgesia. In the recent years, several randomized controlled trials (RCTs) investigated the efficacy of continuous ACB (CACB) versus continuous FNB (CFNB) in patients undergoing knee arthroplasty and no consistent conclusion was made. At present, the advantage of CACB over CFNB is still controversial. Therefore, we attempted to summarize the available RCTs to evaluate CACB versus CFNB on analgesic effect and postoperative movement ability of patients undergoing knee arthroplasty.

## Methods

This systematic review and meta-analysis was conducted and reported in accordance with the Preferred Reporting Items for Systematic Reviews and Meta-analyses (PRISMA) guidelines [12]. The protocol was prospectively registered in the International Prospective Register of Systematic Reviews (PROSPERO CRD42022363756). This study was deemed exempt of ethical approval by the 960^th^ Hospital of PLA Institutional Review Board because only study-level published data were collected.

### Search strategy and study selection

Relevant articles were identified by searching PubMed, Embase and the Cochrane Central Register of Controlled Trials (up to 3 October, 2023) without language restriction. Electronic searches were conducted using the Exploded Medical Subject Headings and appropriate corresponding keywords: “(adductor canal block OR motor sparing knee block OR ACB OR saphenous nerve block) AND (femoral nerve block OR FNB) AND (knee arthroplasty OR knee replacement)”. The findings of the above searches were restricted using a highly sensitive search strategy recommended by the Cochrane Collaboration for identifying randomized controlled trials (RCTs) [13]. The reference lists of RCTs and reviews were checked to identify other potentially eligible trials.

RCTs were included in the present study if they compared CACB with CFNB in patients undergoing knee arthroplasty. The outcomes of pain score and muscle strength were reported in the eligible RCTs. We excluded the literature of non-RCTs, lectures, abstracts, registration schemes, letters and animal experiments. Two authors independently screened the retrieved citations and any disagreement of study selection was arbitrated by a third author.

### Data extraction

Two authors independently extracted the following data from each included trial: year of publication, number of participants, patient characteristics, inclusion and exclusion criteria, type of nerve block, type of anesthesia and study outcomes. Extracted data were entered into a standardized data extraction form and checked by a third author. Any disagreement was resolved by discussion.

The primary outcome was pain score measured by visual analog scale (VAS) or numeric rating scale (NRS) at rest and at motion after knee arthroplasty. Secondary outcomes included opioid consumption, quadriceps muscle strength (maximum voluntary isometric contraction (MIVC) or the Lovett scale), knee function (extension and flexion degrees), functional recovery (TUG test and time to discharge readiness) and risk of falls. The difference in quadriceps muscle strength between the groups was assessed as MVIC as percent of baseline at 24 h postoperative. The Lovett scale is a six-grade scale, in which 0 denotes no muscle contractility and 5 denotes the complete range of motion against gravity, with full resistance.

### Risk of bias assessment

The included RCT_S_ were evaluated for methodological quality according to the method recommended by the Cochrane Collaboration. It mainly includes seven aspects of evaluation: generation of random sequence, allocation concealment, blind method, outcome evaluation, completeness of outcome data, selective reporting and bias of other sources. Based on these criteria, two authors independently rated each included study as low risk, high risk, or unclear. Any disagreement was resolved by discussion or referral to a third author.

### Statistical analysis

All statistical analyses were performed using Review Manager, version 5.4 (RevMan; The Cochrane Collaboration; Oxford, England). Heterogeneity among trials was assessed using the Ι^2^ value. If the Ι^2^ value was < 50%, which represented homogeneity, a fixed-effect model was used. In the case of significant heterogeneity (I^2^ > 50%), a random-effects model was used. Potential sources of heterogeneity were identified by sensitivity analyses performed by omitting one study in each turn and assessing the influence of a single study on the overall pooled estimate. Publication bias was assessed by visually inspecting a funnel plot.

For dichotomous outcomes, risk ratio (RR) with 95% confidence intervals (CIs) were evaluated in accordance with intent-to-treat principles. For continuous outcomes, mean differences (MD) or standardized mean difference (SMD) and 95% CIs were used. P value of less than 0.05 was considered statistically significant.

## Results

### Study identification

**Fig 1:**
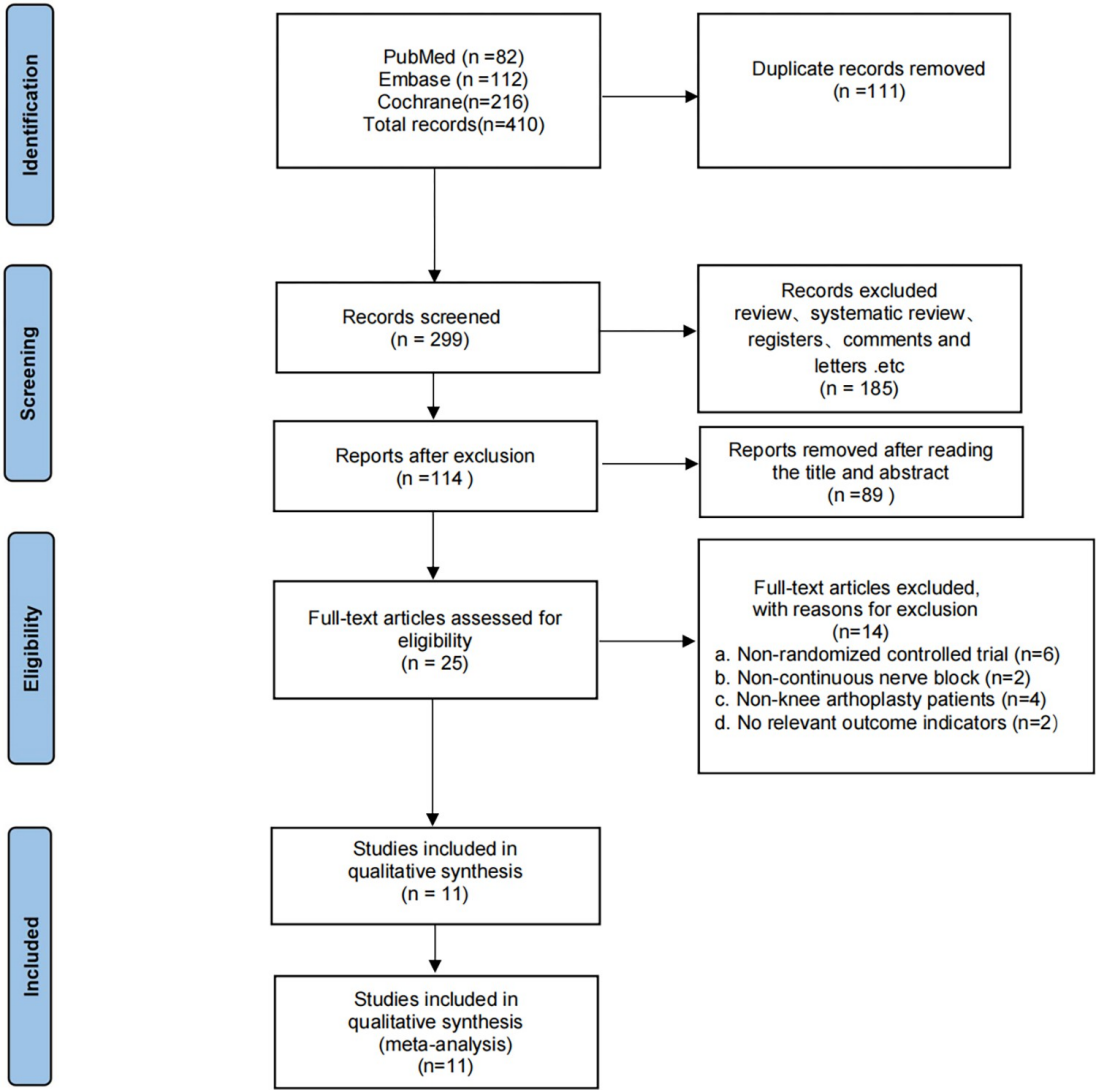
Flowchart of study selection (PRISMA).

Our initial search yielded 410 publications (Fig 1). After screening of the titles and abstracts, 25 potential studies with full-text were retrieved, in which 14 studies did not meet inclusion criteria. Therefore, eleven RCTs [1, 3, 8–10, 14–19] with a total of 748 patients were eligible for inclusion in the present study.

The included trials were published between 2013 and 2022 with sample sizes ranging from 30 to 151. Among the included trials, three [1, 3, 16] were conducted in North America, four [9, 10, 17, 19] in Asia, three [8, 14, 18] in Europe and one [15] in Australia. Three trials [8, 15, 16] were multicenter studies. Ten trials [1, 3, 8, 9, 14–19] were published in English and one [10] in Chinese. Two trials [1, 16] were conducted by the same study team on different patients. Ten studies [3, 8–10, 14–19] recruited patients undergoing total knee arthroplasty and the remaining study [1] recruited patients undergoing unicompartment knee arthroplasty. Chronic preoperative opioid use was an exclusion criteria in seven included studies [1, 3, 8, 10, 15–17]. All the eleven [1, 3, 8–10, 14–19] studies used other analgesic interventions (periarticular infiltrates, oral analgesics or other nerve block modalities). Table 1 provided the baseline characteristics of all participants included in the present review.

**Table 1:** Baseline characteristics of included studies.

Study ID	Country	Cases	Age (mean)	Gender (male%)	BMI (mean)	Intervention technique		Anesthesia	Time	Follow up	Outcomes
		CACB/CFNB	CACB/CFNB	CACB/CFNB	CACB/CFNB	CACB	CFNB				
Jæger, 2013 [8]	Denmark	23/27	70/66	21.7/51.9	26.9/28.7	Halfway between ASIS and the patella with US; Initially 30 ml of 0.5% ropivacaine, 0.2% ropivacaine 8 ml/h until 24 h	Parallel to the inguinal crease with US; Initially 30 ml of 0.5% ropivacaine, 0.2% ropivacaine 8 ml/h until 24 h	Spinal	Postoperative	24h	Muscle tests of the quadriceps, pain, ROM, TUG, opioid dose
Shah, 2014 [17]	India	48/50	68.3/65.9	27.1/28	29.5/ 30.5	Halfway between the ASIS and the patella, at the midthigh level with US; 30 ml ropivacaine 0.75% with ropivacaine 0.2% 30 ml/4 h until 8:00 am on POD 1	Just below the inguinal crease with the nerve stimulator; 30 ml ropivacaine 0.75% with ropivacaine 0.2% 30 ml/4 h until 8:00 am on POD 1	Spinal	Postoperative	48h	Ambulation ability (TUG, 10-m walk, 30 s chair test), staircase competency, ambulation distance, pain scores, opioid consumption, length of hospital stay
Zhang, 2014 [9]	China	30/30	63.7/61.9	20/26	25.1/24.0	8–12 cm below the inguinal crease with US; 20 ml of 0.33% ropivacaine with 0.2% ropivacaine 5 ml/ h until 48 h	1.0 cm below the Inguinal crease with the nerve stimulator; 20 ml of 0.33% ropivacaine with 0.2% ropivacaine 5 ml/ h until 48 h	Spinal-epidural	Postoperative	48h	Pain, quadriceps strength, complications
Machi, 2015 [16]	America	39/41	67/ 66	41/34	30/ 29	Midpoint between the ASIS and the patella with US; 30 ml of lidocaine 2% with 0.2% ropivacaine 6 ml/ h until the morning of POD 3	at the inguinal crease with US; 30 ml of lidocaine 2% with 0.2% ropivacaine 6 ml/ h until the morning of POD 3	Spinal or GA	Preoperative	72h	Pain, opioid dose, ambulation distance, TUG, quadriceps, knee scores
Sztain, 2015 [1]	America	15/15	70/68	53/53	28/30	Midpoint between the ASIS and the patella with US; 30 ml of lidocaine 2% with 0.2% ropivacaine 6 ml/ h until 48 h	at the inguinal crease with US; 30 ml of lidocaine 2% with 0.2% ropivacaine 6 ml/ h until 48 h	Spinal or GA	Preoperative	72h	Pain, opioid dose, ambulation distance, TUG, quadriceps, knee scores
Wiesmann, 2016 [18]	Germany	21/21	72/ 66	43/43	29/32	Guided by US and nerve stimulator. 20 cm proximal to the cranial margin of the patella; 15 ml of 0.375% ropivacaine with 0.2% ropivacaine 6 ml/ h for 3 days	Guided by US and nerve stimulator. 15 ml of 0.375% ropivacaine with 0.2% ropivacaine 6 ml/ h for 3 days	GA	Preoperative	72h	TUG, opioid, pain, quadriceps, ambulation distance
Elkassabany, 2016 [3]	America	31/31	63/65	29/39	31/32	US; 20 ml of 0.5% ropivacaine with 0.2% ropivacaine 8 ml/ h until 7:00 am on POD 1	US; 20 ml of 0.5% ropivacaine with 0.2% ropivacaine 8 ml/ h until 7:00 am on POD 1	Spinal or GA	Preoperative	7 days	Fall risk, muscle tests of the quadriceps, TUG, ambulation distance, pain, opioid dose, patient-oriented outcomes
Zhao, 2017 [10]	China	20/20	65.0/62.6	15/20	27.8/28.9	Midpoint between the ASIS and the patella with US; 20 ml of 0.2% ropivacaine with 0.2% ropivacaine 5 ml/ h for 48 h	at the inguinal crease with US; 20 ml of 0.2% ropivacaine with 0.2% ropivacaine 5 ml/ h for 48 h	Spinal-epidural	Postoperative	48h	Pain, Quadriceps muscle strength, Adverse reactions caused by the addition of opioids
Borys, 2019 [14]	Poland	43/42	67.3/68.8	18.6/19	31.9/30.8	US; 0.2% ropivacaine 5 ml/ h	US; 0.2% ropivacaine 5 ml/ h	Spinal	Postoperative	Until discharge	Total number of morphine uses, Pain, quadriceps muscle strength, knee extension, sitting, standing upright, and walking.
Chuan, 2019 [15]	Australia	75/76	66/68	51/53	31.6/32.9	2 cm distal to the apex of the femoral triangle with US; 15 ml of 0.2% ropivacaine with 0.2% ropivacaine 8 ml/ h	midpoint of the femoral triangle with US; 15 ml of 0.2% ropivacaine with 0.2% ropivacaine 8 ml/ h	Spinal	immediately after spinal block or at the end of surgery, depending on the policy at each recruitment site.	48h	TUG, opioid consumption, pain
Siddiqui, 2022 [19]	India	25/25	63.6/63.5	44/32	NA	Combined with IPACK block; 20 ml of 0.2% ropivacaine with 0.2% ropivacaine 3 ml/ h	Combined with IPACK block; 20 ml of 0.2% ropivacaine with 0.2% ropivacaine 3 ml/ h	GA	Postoperative	24h	Movement of the operated limb, TUG, pain, opioid, patient satisfaction

BMI, body mass index; CACB, continuous adductor canal block; CFNB, continuous femoral nerve block; ROM, range of motion; TUG, time up and go; US, Ultrasound; POD, post operation day; GA, general Anesthesia; NA, not available; IPACK, interspace between the popliteal artery and capsule of the posterior knee block; ASIS, anterior superior iliac spine.

**Fig 2:**
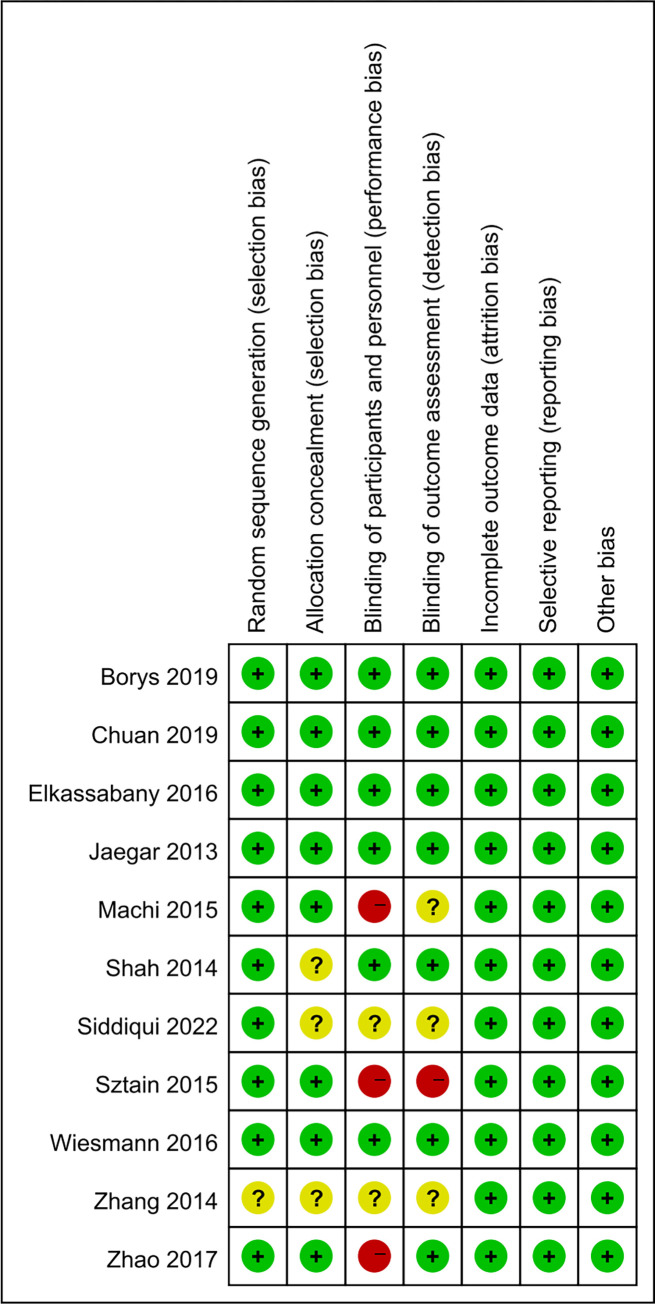
Risk of bias.

Among all the included trials, randomized sequence and allocation sequence concealment were conducted adequately in ten [1, 3, 8, 10, 14–19] and eight [1, 3, 8, 10, 14–16, 18] studies, respectively. Blinding of patients and outcome assessment was clearly stated in six [3, 8, 14, 15, 17, 18] and seven [3, 8, 10, 14, 15, 17, 18] studies, respectively. All studies [1, 3, 8–10, 14–19] had low risks of loss of follow-up or withdrawal, selection reports and other types of bias. In addition, each of the included studies reported comparable baseline characteristics. Finally, poor randomization/assignment hiding/blinding resulted in a high risk of bias in three [1, 10, 16] studies and an unclear risk of bias in six [1, 9, 10, 16, 17, 19] studies. A summary of the risk of bias of the included studies was shown in Fig 2.

### Meta-analysis results

The primary outcome was pain score measured at rest and at motion after knee arthroplasty. Among the included eleven studies, five [10, 16–19], nine [3, 8–10, 14, 16–19] and seven [3, 9, 10, 14, 16–18] studies reported pain score at rest at 12 h, 24 h and 48 h postoperative, respectively. Three [10, 17, 18], five [8–10, 17, 18] and four [9, 10, 17, 18] studies reported pain score at motion at 12 h, 24 h and 48 h postoperative, respectively. There was not an outcome for which all the eleven included studies reported results. Therefore, we conducted meta-analyses of different combinations of studies reporting endpoints for pain at rest and at motion at different time points.

#### Pain score at rest after knee arthroplasty

**Fig 3:**
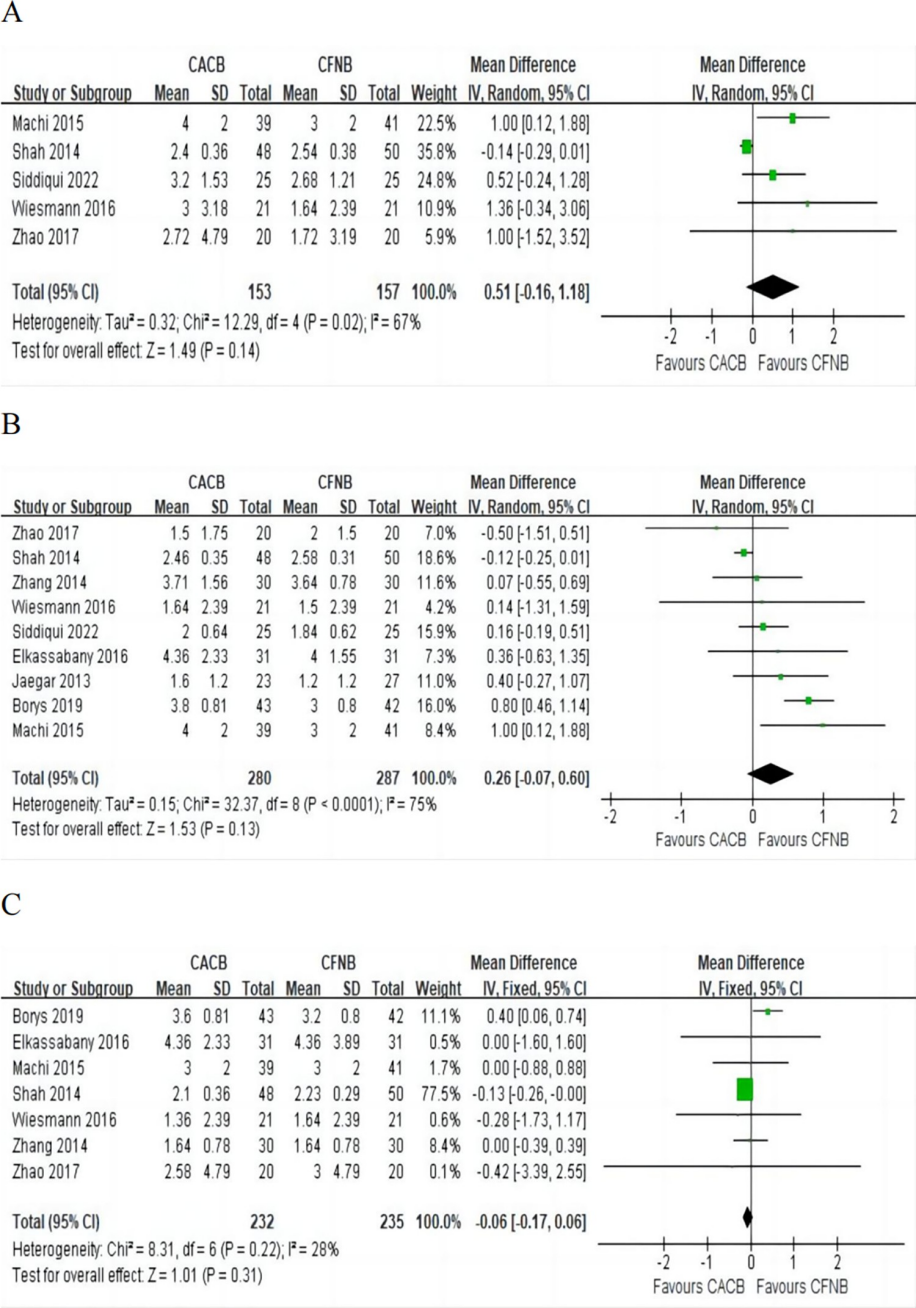
Forest plot of the pain score at rest. A, pain scores at rest at 12 h, I^2^ = 67%; B, pain scores at rest at 24 h, I^2^ = 75%; C, pain scores at rest at 48 h, I^2^ = 28%.

Pain score at 12 h: This outcome was reported in five [10, 16–19] studies involving 310 patients, including 153 in the CACB group and 157 in the CFNB group. Pooled analysis indicated that there was no significant difference in pain score at 12 h at rest between the two interventions (MD = 0.51, 95% CI (-0.16, 1.18), P = 0.14) (Fig 3) with significant heterogeneity. Sensitivity analyses indicated that the heterogeneity was best solved after excluding the study by Shah et al and the resting pain score at 12 h were higher in the CACB group than that in the CFNB group (MD = 0.80, 95% CI (0.27, 1.34), P = 0.003).

Pain score at 24 h: Data from nine [3, 8–10, 14, 16–19] studies involving 567 patients was available for the outcome. Pooled analysis suggested that there was no significant difference in pain score at 24 h at rest between the CACB group and the CFNB group (MD = 0.26, 95% CI (-0.07, 0.60), P = 0.13) (Fig 3) with significant heterogeneity. Sensitivity analyses indicated that the heterogeneity was best solved after excluding the study by Borys et al and the effect estimate remained nonsignificant (MD = 0.10, 95% CI (-0.14, 0.35), P = 0.41).

Pain score at 48 h: The pain score at 48 h at rest after knee arthroplasty was reported in seven [3, 9, 10, 14, 16–18] studies involving 467 patients, including 232 in the CACB group and 235 in the CFNB group. No significant difference was found between the two groups (MD = -0.06, 95% CI (-0.17, 0.06), P = 0.31) (Fig 3) with no significant heterogeneity.

#### Pain score at motion after knee arthroplasty

**Fig 4:**
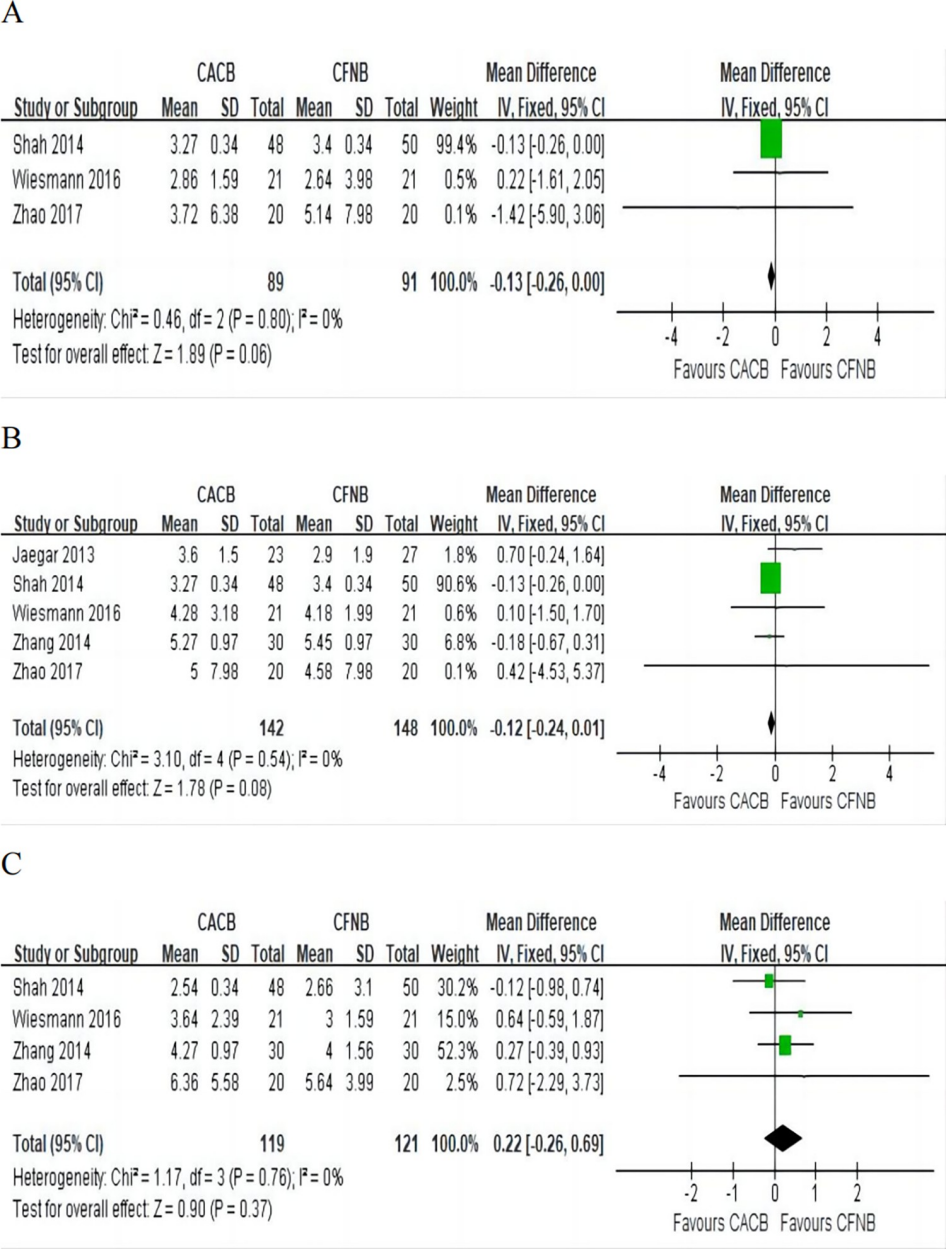
Forest plot of the pain score at motion. A, pain scores at motion at 12 h, I^2^ = 0%; B, pain scores at motion at 24 h, I^2^ = 0%; C, pain scores at motion at 48 h, I^2^ = 0%.

Pain score at 12 h: This outcome was reported in three [10, 17, 18] studies involving 180 patients, including 89 in the CACB group and 91 in the CFNB group. Pooled analysis indicated that there was no significant difference in pain score at 12 h at motion between the two interventions (MD = -0.13,95% CI (-0.26, 0.00), P = 0.06) (Fig 4) with no significant heterogeneity.

Pain score at 24 h: Data from five [8–10, 17, 18] studies involving 290 patients was available for the outcome. Pooled analysis suggested that there was no significant difference in pain score at 24 h at motion between the CACB group and the CFNB group (MD = -0.12, 95% CI (-0.24, 0.01), P = 0.08) (Fig 4) with no significant heterogeneity.

Pain score at 48 h: The pain score at 48 h at motion after knee arthroplasty was reported in four [9, 10, 17, 18] studies involving 240 patients, including 119 in the CACB group and 121 in the CFNB group. No significant difference was found between the two groups (MD = 0.22, 95% CI (-0.26, 0.69), P = 0.37) (Fig 4) with no significant heterogeneity.

#### Opioid consumption

Opioid consumption at 24 h: This outcome was reported in four [3, 8, 15, 19] studies with a total of 313 patients, including 154 in the CACB group and 159 in the CFNB group. Pooled analysis indicated that there was no significant difference in opioid consumption at 24 h between the two groups (SMD = 0.22, 95% CI (-0.21, 0.64), P = 0.32) (S1 Fig) with significant heterogeneity.

Opioid consumption at 48 h: Data from two [3, 15] studies involving 213 patients was available for the outcome. No significant difference was found between the two groups (SMD = -0.01,95% CI (-0.28, 0.26), P = 0.94) (S1 Fig) with no significant heterogeneity.

#### Quadriceps muscle strength

**Fig 5:**
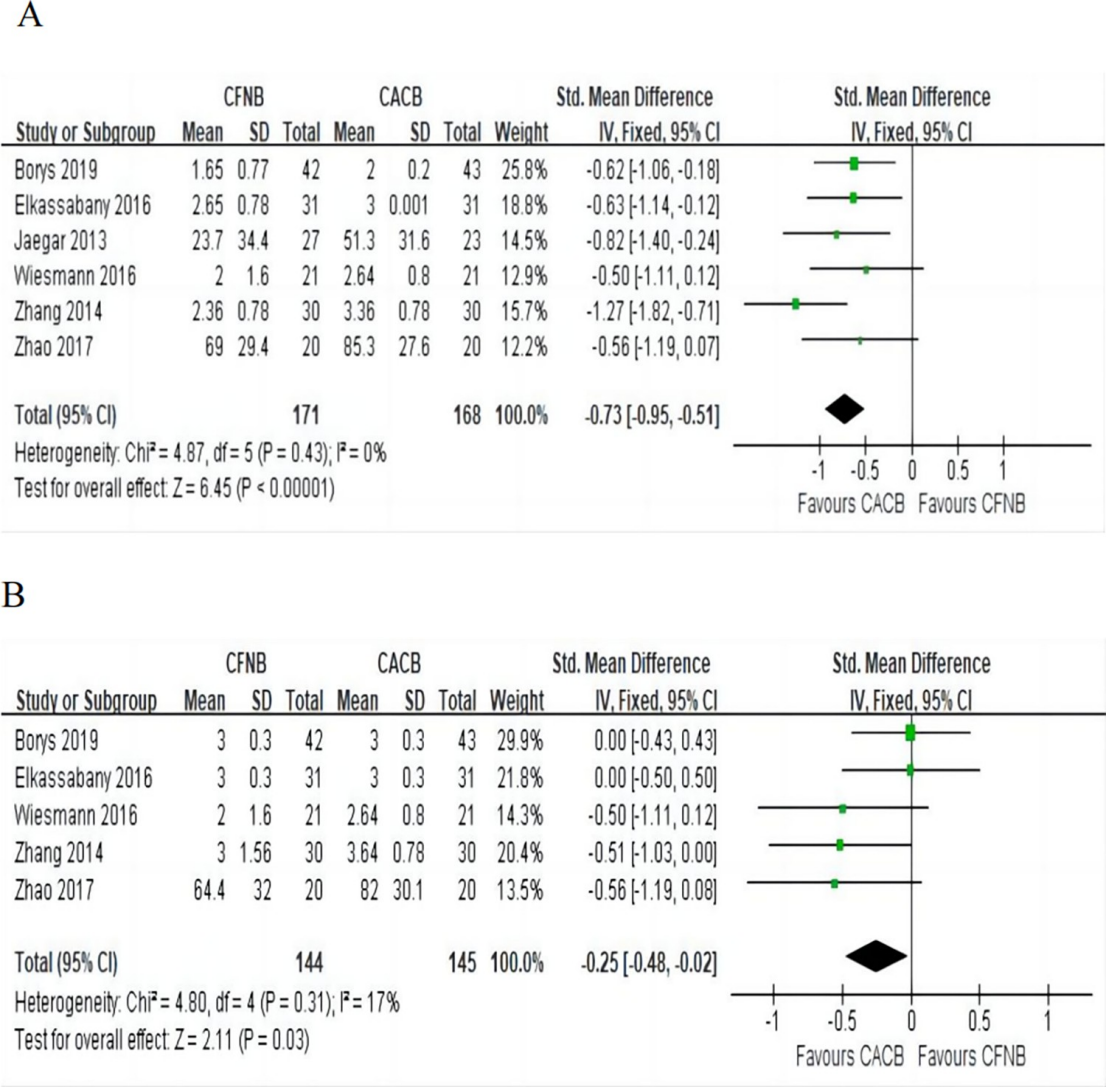
Forest plot of the quadriceps muscle strength. A, quadriceps muscle strength at 24 h, I^2^ = 0%; B, quadriceps muscle strength at 48 h, I^2^ = 17%.

Quadriceps muscle strength at 24 h: This outcome was reported in six [3, 8–10, 14, 18] studies in 339 patients, including 168 in the CACB group and 171 in the CFNB group. Pooled analysis suggested that the quadriceps muscle strength of the CACB group was better than that of the CFNB group (SMD = -0.73, 95% CI (-0.51, -0.95), P<0.0001) (Fig 5) with no significant heterogeneity.

Quadriceps muscle strength at 48 h: Data from five [3, 9, 10, 14, 18] studies involving 289 patients was available for the outcome. Pooled analysis suggested that the quadriceps muscle strength in the CACB group was better than that in the CFNB group (SMD = -0.25, 95% CI (-0.48, -0.02), P = 0.03) (Fig 5) with no significant heterogeneity.

#### Knee function

Knee extension degrees: This outcome was reported in two [1, 16] studies with a total of 110 patients, including 54 in the CACB group and 56 in the CFNB group. Pooled analysis indicated that there was no significant difference in knee extension degrees between the two groups (MD = -1.94, 95% CI (-5.86, 1.98), P = 0.33) (S2 Fig) with significant heterogeneity.

Knee flexion degrees: Data from three [1, 16, 17] studies involving 208 patients was available for the outcome. Pooled analysis showed that there was no significant difference in knee flexion degrees between the two groups (MD = 0.33, 95% CI (-7.22, 7.87), P = 0.93) (S2 Fig) with significant heterogeneity.

#### Functional recovery

**Fig 6:**
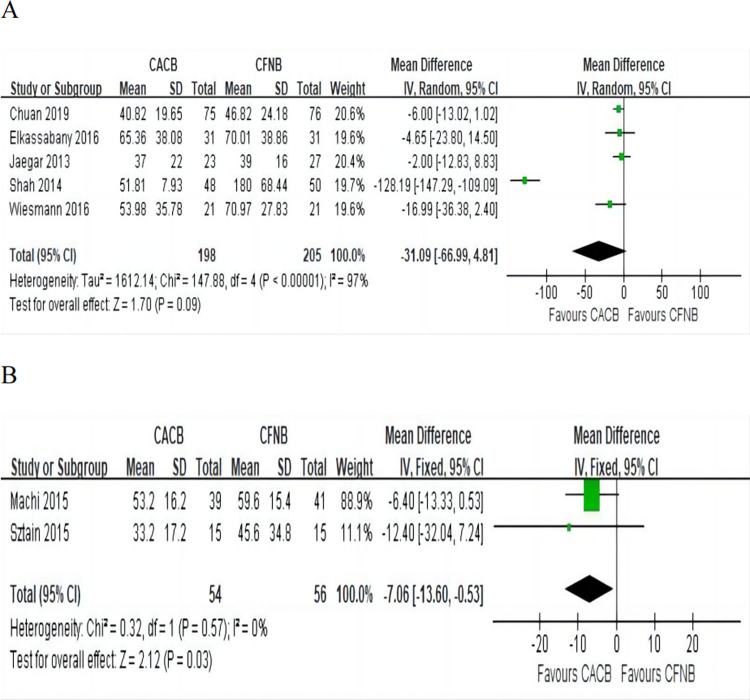
Forest plot of the functional recovery. A, TUG test, I^2^ = 97%; B, Time to discharge readiness, I^2^ = 0%.

TUG test: TUG test was used to assess patients’ walking ability and functional recovery status. This outcome was reported in five [3, 8, 15, 17, 18] studies involving 403 patients, including 198 in the CACB group and 205 in the CFNB group. Pooled analysis showed that there was no significant difference in TUG test between the two groups (MD = -31.09, 95% CI (-66.99, 4.81), P = 0.09) (Fig 6) with significant heterogeneity.

Time to discharge readiness: Data from two [1, 16] studies involving 110 patients was available for the outcome. Pooled analysis indicated that the time to discharge readiness in the CACB group was shorter than that in the CFNB group (MD = -7.06, 95% CI (-13.60, -0.53), P = 0.03) (Fig 6) with no significant heterogeneity.

#### Risk of falls

This outcome was reported in four [1, 8, 16, 18] studies involving 202 patients, including 98 in the CACB group and 104 in the CFNB group. A total of seven fall events were reported in the CFNB group, while only two fell events occurred in the CACB group. Pooled analysis suggested that patients in the CACB group had a lower risk of falls than those in the CFNB group, but the difference was not statistically significant (RR = 0.38, 95% CI (0.11, 1.39), P = 0.15) (S3 Fig) with no significant heterogeneity.

### Publication bias

The scatter of results on funnel plot was broadly symmetric on visual inspection, suggesting an absence of serious publication bias in this meta-analysis (S4 Fig).

## Discussion

In the present meta-analysis, both CACB and CFNB showed the same degree of pain relief at rest and at motion and opioid consumption at 12 h, 24 h and 48 h in patients undergoing knee arthroplasty. In addition, there was no significant difference in knee extension and flexion, TUG test, or risk of falls between the CACB group and the CFNB group. Compared with CFNB, CACB preserved the quadriceps muscle strength better and significantly shortened the discharge readiness time, suggesting that patients undergoing CACB had earlier functional recovery after surgery.

Although the results of our meta-analysis were in line with the results of a previous meta-analysis by Zhang et al [20], differences between the two meta-analysis should be noted. In Zhang et al’s meta-analysis, seven RCTs and four retrospective studies were included, thereby producing a potential for selection bias. In our updated meta-analysis, the inclusion criteria were strictly restricted to RCTs, and thus eleven RCTs with a total of 748 patients were finally included, providing the latest and more robust evidence to evaluate the effectiveness of CACB versus CFNB in postoperative analgesia and early rehabilitation of patients with knee arthroplasty. In addition, a meta-analysis by Hasabo et al [21] investigating ACB versus FNB in patients undergoing total knee arthroplasty included RCTs and cohort studies involving both single-injection and continuous nerve block and showed similar results with our meta-analysis. Likewise, a systematic review by Karkhur et al [22] suggested that VAS scores for pain and consumption of opioids were similar between ACB and FNB, indicating comparable efficacy.

The greater preservation of quadriceps strength and ambulation activity in patients administered ACB, as compared to FNB, is an attribute of the mechanism of the block [22]. Anatomically, the femoral nerve is located on the lateral side of the femoral artery below the inguinal ligament and enters the thigh where it divides into terminal branches. Therefore, FNB is usually performed at or below the inguinal ligament, leading to nerve block in the entire front of the upper thigh and the medial leg to the medial femoral malleolus. Successful blockade promotes weakening of quadriceps muscle strength and may increases the occurrence of falls, which may be not conducive to the postoperative functional exercise of patients undergoing knee arthroplasty. However, the main target of ACB is the saphenous nerve. The saphenous nerve is the only cutaneous nerve in the posterior branch of the femoral nerve, and it is a pure sensory nerve. ACB is almost a pure sensory nerve block with little effect on the strength of the quadriceps femoris muscle. Jaeger et al [23] found that in healthy volunteers saphenous nerve block only reduced the quadriceps muscle strength by 8%, while FNB reduced the muscle strength by 49%. Our meta-analysis suggested that CACB had advantage in retaining quadriceps muscle strength than CFNB in patients undergoing knee arthroplasty, which is basically consistent with the results of previous studies [3, 9].

Previous studies have shown that FNB has some serious complications, such as decreased quadriceps muscle strength and increased clinical falls [24]. Turbitt et al reported a higher rate of falls in patients treated with CFNB [25]. In contrast, CACB only results in weakness of the vastus medialis, thereby theoretically significantly reducing the chance of falling. Several studies have shown that there are no fall incidents in patients with CACB after total knee replacement [3, 14–16]. In our meta-analysis, four RCTs involving a total of 202 patients reported the outcome of risk of falls and patients in the CACB group had a lower risk of falls than those in the CFNB group, but the difference was not statistically significant, which was consistent with a previous meta-analysis by Zhang et al [20]. Due to the limited number of RCTs and patients included, our meta-analysis was underpowered for this important outcome and should not be taken as supporting or refuting the potential benefits of CACB on falls.

The present meta-analysis had several limitations. First, considerable heterogeneity existed in population characteristics, different regimens of CACB and CFNB used and different types of anesthesia (general or spinal) across the included trials. Second, sensitivity analyses indicated that the resting pain score at 12 h postoperative were higher in the CACB group than that in the CFNB group after excluding the study by Shah et al. Due to the limited number of studies included, caution should be taken when interpreting the result of sensitive analysis and more high-quality studies with large samples were needed in the future. Third, the patient follow-up period was short in the included studies and only short-term outcomes after knee arthroplasty were analyzed in the present meta-analysis. The long-term effect of CACB versus CFNB on pain control and knee joint function recovery should be evaluated in the future. Four, the geographic regions covered in the current meta-analysis included North America (United States), Europe (Germany, Denmark and Poland), Asia (China and India) and Oceania (Australia). Therefore, our results limited generalizability to other regions (for example, Latin America and West Asia).

## Conclusion

Compared with CFNB, CACB has similar effects on pain relief both at rest and at motion and opioid consumption for patients undergoing knee arthroplasty, while CACB is better than CFNB in preserving quadriceps muscle strength and shortening the discharge readiness time. Thus, CACB has the potential to be an alternative to CFNB for pain relief in patients undergoing knee arthroplasty.

## Supporting information

S1 Checklist PRISMA 2020 checklist. (DOCX)

S1 Fig Forest plot of the opioid consumption. A, Opioid consumption at 24 h, I^2^ = 69%; B, Opioid consumption at 48 h, I^2^ = 0%.
(TIF)

S2 Fig Forest plot of the knee function. A, Knee extension degrees, I^2^ = 86%; B, Knee flexion degrees, I^2^ = 74%.
(TIF)

S3 Fig Forest plot of the risk of falls. Risk of falls, I^2^ = 0%.
(TIF)

S4 Fig Funnel plot of the publication bias. (TIF)
