# Supplementary figures and images for: Continuous adductor canal block versus continuous femoral nerve block for postoperative pain in patients undergoing knee arthroplasty: An updated meta-analysis of randomized controlled trials
Source: PLoS One. 2024 Aug 1;19(8):e0306249. doi: 10.1371/journal.pone.0306249 (PMC11293650; doi:10.1371/journal.pone.0306249)

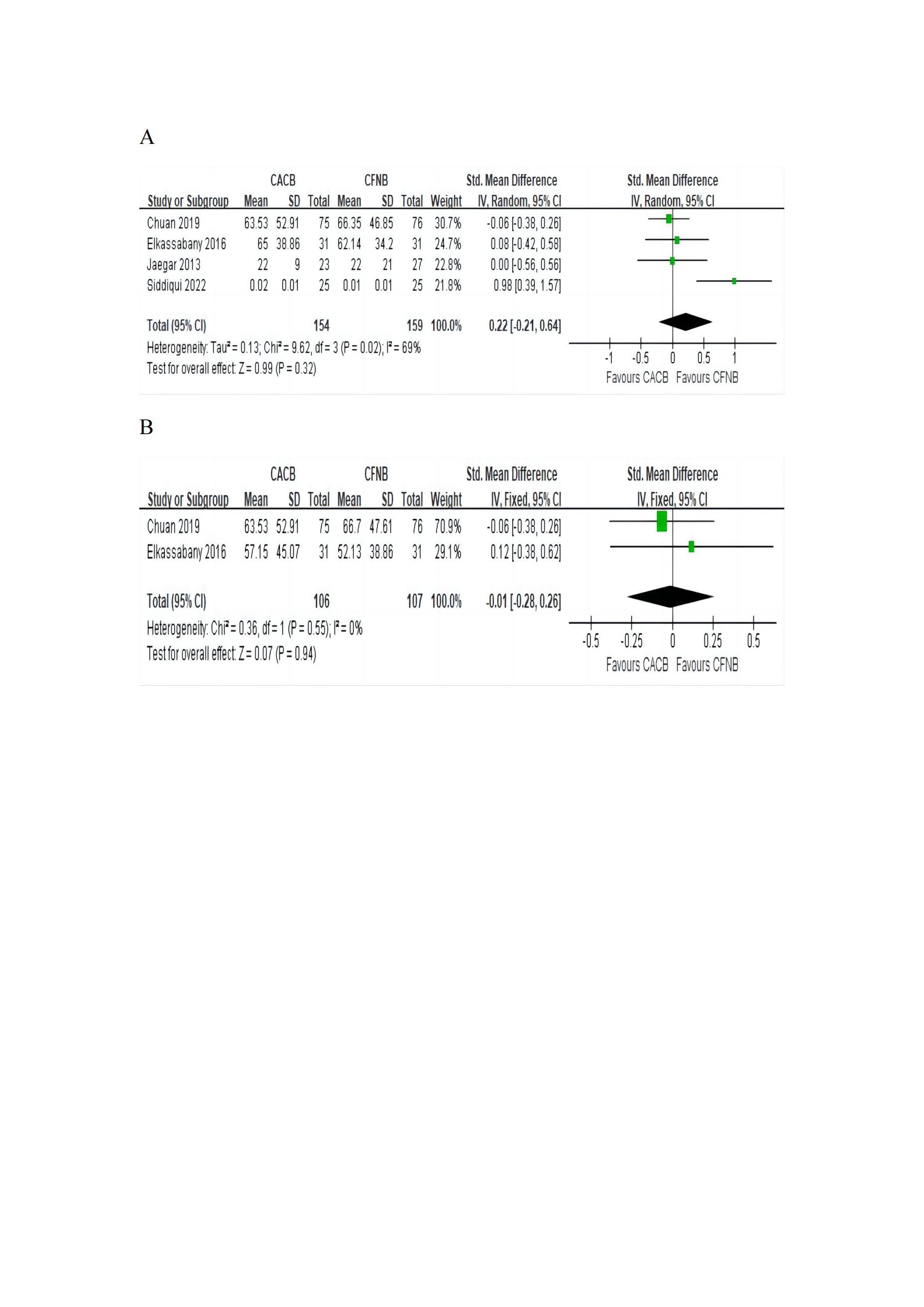

Supplement: S1 Fig — A, Opioid consumption at 24 h, I2 = 69%; B, Opioid consumption at 48 h, I2 = 0%. (TIF) [file pone.0306249.s002.tif]

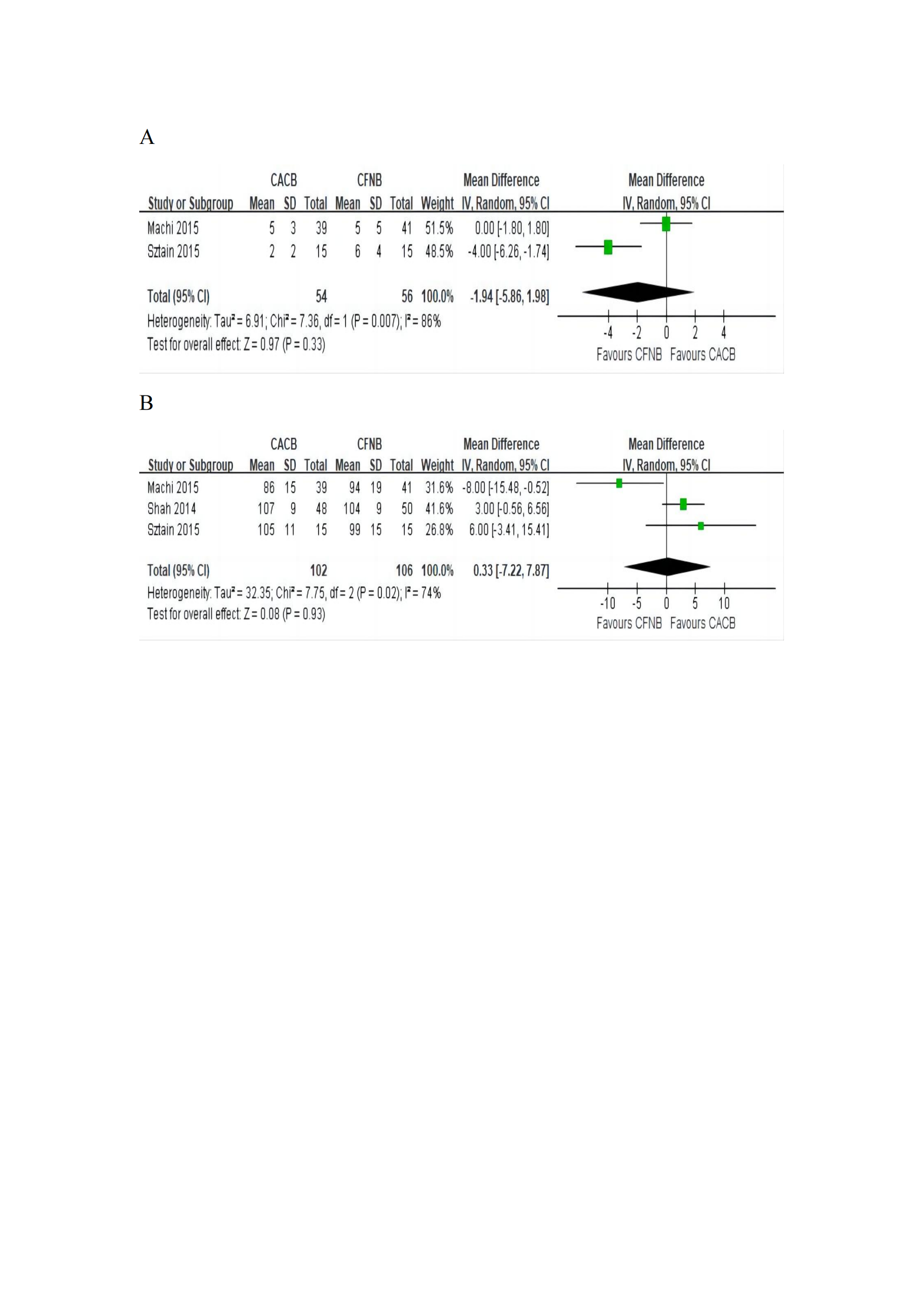

Supplement: S2 Fig — A, Knee extension degrees, I2 = 86%; B, Knee flexion degrees, I2 = 74%. (TIF) [file pone.0306249.s003.tif]

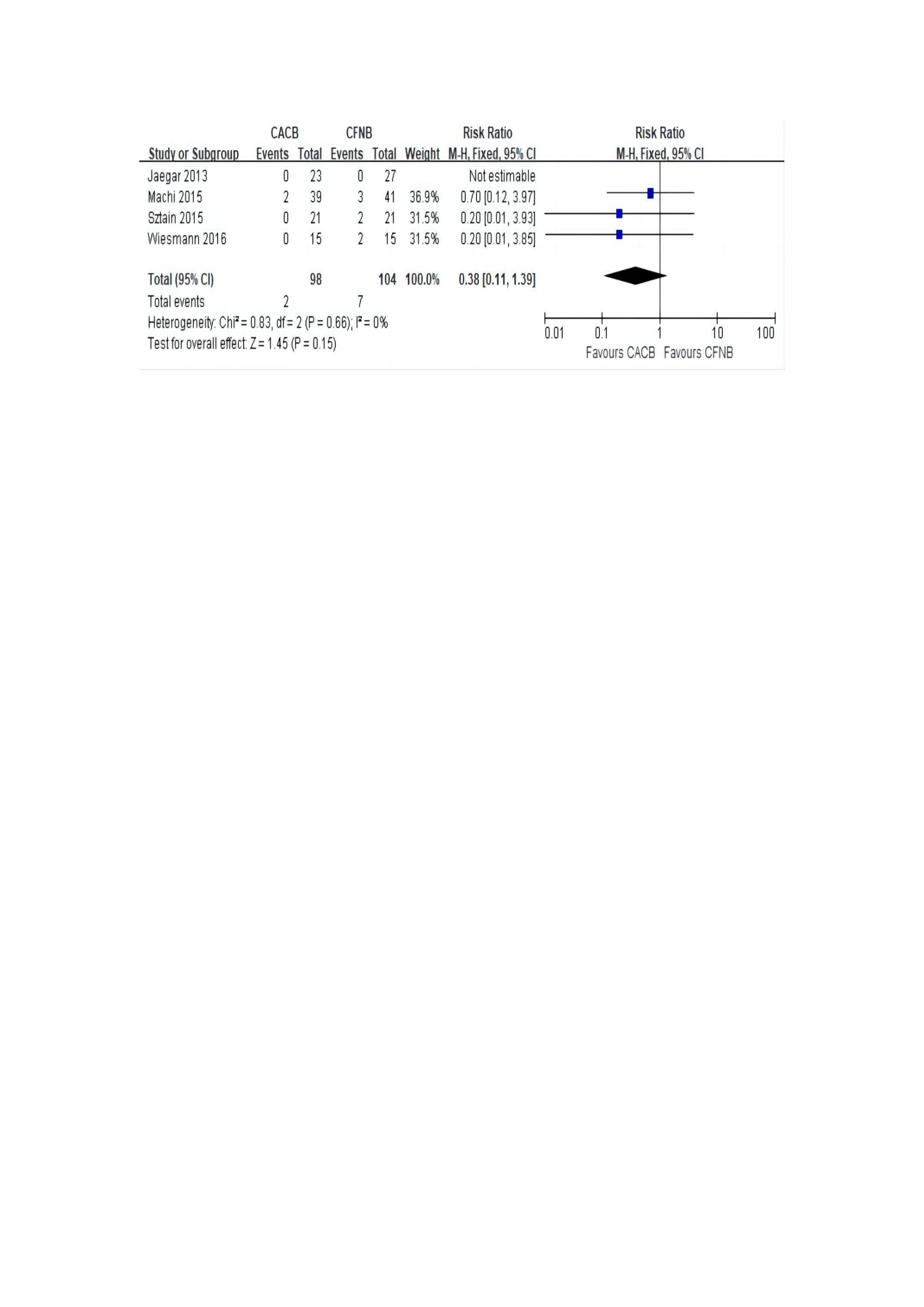

Supplement: S3 Fig — Risk of falls, I2 = 0%. (TIF) [file pone.0306249.s004.tif]

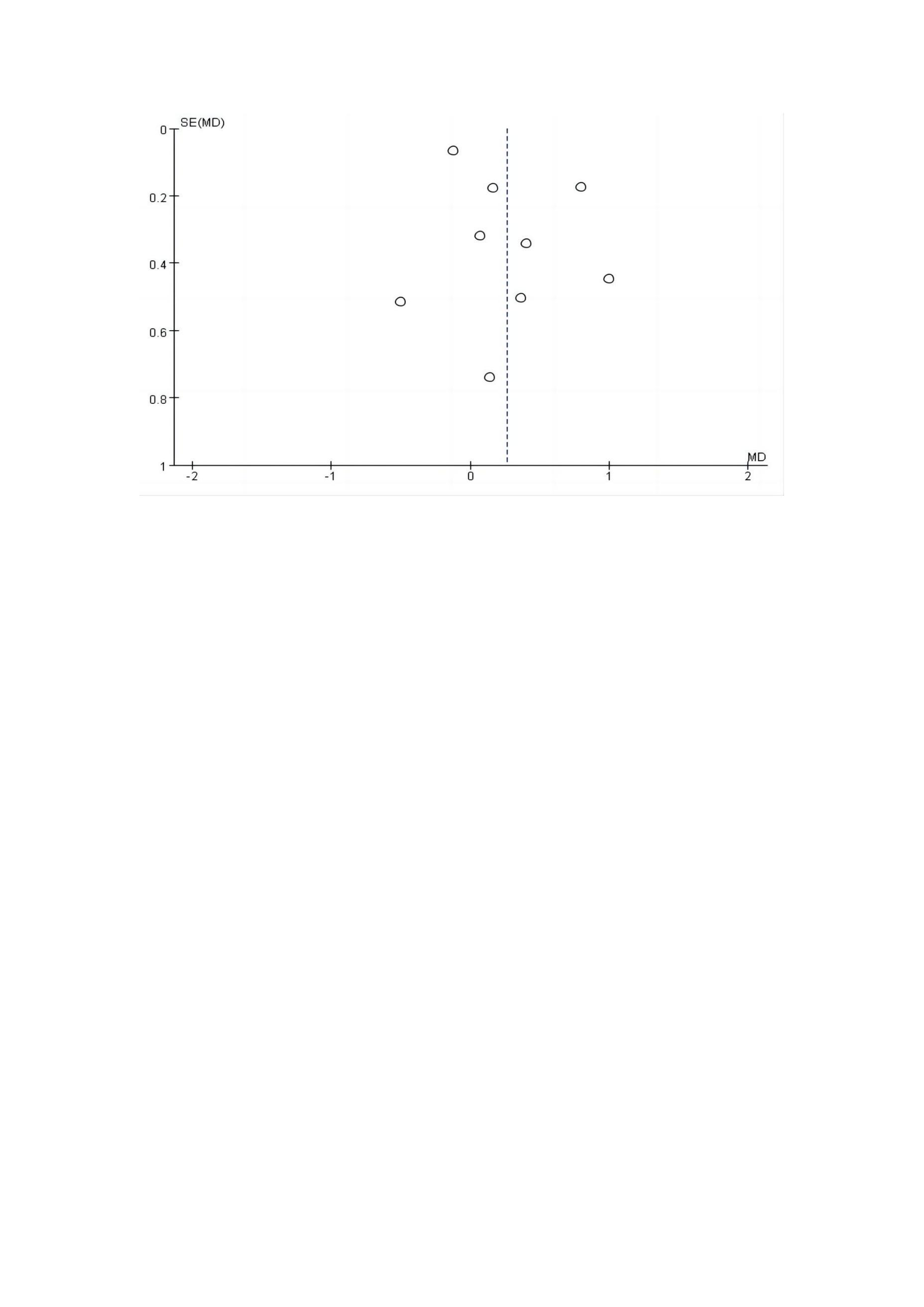

Supplement: S4 Fig — (TIF) [file pone.0306249.s005.tif]
